# Expansion of cytochrome P450 and cathepsin genes in the generalist herbivore brown marmorated stink bug

**DOI:** 10.1186/s12864-017-4281-6

**Published:** 2018-01-18

**Authors:** Raman Bansal, Andy Michel

**Affiliations:** Department of Entomology, Ohio Agricultural Research and Development Center, The Ohio State University, 1680 Madison Avenue, Wooster, OH 44691 USA

**Keywords:** *Halyomorpha halys*, Generalist, Invasion, Herbivory, Adaptation

## Abstract

**Background:**

The brown marmorated stink bug (*Halyomorpha halys*) is an invasive pest in North America which causes severe economic losses on tree fruits, ornamentals, vegetables, and field crops. The *H. halys* is an extreme generalist and this feeding behaviour may have been a major contributor behind its establishment and successful adaptation in invasive habitats of North America. To develop an understanding into the mechanism of *H. halys*’ generalist herbivory, here we specifically focused on genes putatively facilitating its adaptation on diverse host plants.

**Results:**

We generated over 142 million reads via sequencing eight RNA-Seq libraries, each representing an individual *H. halys* adult. The de novo assembly contained 79,855 high quality transcripts, totalling 39,600,178 bases. Following a comprehensive transcriptome analysis, *H. halys* had an expanded suite of cytochrome P450 and cathepsin-L genes compared to other insects. Detailed characterization of P450 genes from the CYP6 family, known for herbivore adaptation on host plants, strongly hinted towards *H. halys*-specific expansions involving gene duplications. In subsequent RT-PCR experiments, both P450 and cathepsin genes exhibited tissue-specific or distinct expression patterns which supported their principal roles of detoxification and/or digestion in a particular tissue.

**Conclusions:**

Our analysis into P450 and cathepsin genes in *H. halys* offers new insights into potential mechanisms for understanding generalist herbivory and adaptation success in invasive habitats. Additionally, the large-scale transcriptomic resource developed here provides highly useful data for gene discovery; functional, population and comparative genomics as well as efforts to assemble and annotate the *H. halys* genome.

**Electronic supplementary material:**

The online version of this article (10.1186/s12864-017-4281-6) contains supplementary material, which is available to authorized users.

## Background

Invasive species are a severe threat to ecological and environmental diversity and sustainability. In agroecosystems, invasive species are thought to be responsible for ~$62 billion worth of economic impacts related to crop production [1, 2]. Despite their seeming ubiquity, invasive species present an ecological and evolutionary paradox, in that the founding populations are often small in number and may not harbor substantial genetic diversity for successful establishment [3, 4]. There have been many hypotheses and much evidence to support how invasive species overcome this genetic diversity constraint, including a lack of natural enemies in the invaded habitat, high reproductive output, and wide-ranging dispersal ability.

Herbivorous insects are notorious invaders, and one additional hypothesis may be the interaction between insects and their host plants, including host breadth. While there are some invasive species that are specialists (e.g. several aphid species, see [5]), the ecological constraint of host plant selection in invaded areas is likely much less with generalists which have a myriad of suitable hosts available. Nonetheless, insect-plant interactions involve complex genetic and molecular processes, and are likely still dependent on genetic diversity.

Although a comprehensive understanding into the underlying mechanisms of generalist herbivory remains elusive [6–9], molecular investigations into insect-plant interactions have revealed a few important genes and gene families. For example, cytochrome P450 enzymes have been found to mediate adaptation of several insect herbivores on host plants [10, 11]. Through their monooxygenase activity, P450s can readily catalyze the detoxification of chemicals [plant secondary metabolites (PSMs) or allelochemicals] which are detrimental to the herbivore’s survival and reproduction [12, 13]. P450 genes comprise one of the largest gene families in insects and are distributed into four well-supported clades called CYP clans (clan 2, mitochondrial clan, clan 3, and clan 4); members of CYP3 clan are usually implicated in herbivore adaptation on plant hosts [13]. A large diversity of insect P450 genes resulting from species- or lineage-specific expansions (e.g. duplication followed by divergence) has been observed [14]. The number of P450 genes in insect genomes (i.e. CYPome size) is highly variable but, in general, generalist herbivores possess a significantly larger repertoire of P450 genes than a specialist, presumably to cope with diverse and unpredictable host challenges [15]. For example, the generalist aphid *Myzus persicae*, which feeds on more than 100 species in 40 different plant families, has at least 40% more P450 genes compared to the specialist aphid *Acyrthosiphon pisum* [16].

Another example of a gene superfamily involved in host plant adaptation is cathepsins. These are proteolytic enzymes employed by insects to defend against plant proteases and plant protease inhibitors that target insect gut tissues and digestive proteins (reviewed in [17]). Additionally, insect cathepsins can break down a variety of protein fractions in their dietary intake, thus facilitating the survival and adaptation on diverse plant hosts [18]. The role of cathepsins as digestive proteases is highly significant in herbivorous heteropterans such as stink bugs because their diet contains complex macromolecules (as opposed to far the simpler diet of phloem feeding hemipterans like aphids and leafhoppers) which need to be broken down for absorption from the gut [19]. Further, in addition to performing roles within the gut tissues, cathepsins remove harmful proteases and protease inhibitors and perform the extra oral digestion following their secretion from the salivary glands into the host plant substrate [20].

The brown marmorated stink bug, *Halyomorpha halys* (Heteroptera: Pentatomidae) is a generalist herbivore which is thought to have invaded North America in the 1990s after being initially observed in the United States (U.S.) in Allentown, PA around 1996 [21]. The *H. halys* invasion occurred from its native range in Asia, and most likely China [22]. Following the invasion, *H. halys* has spread rapidly across North America and, as of January 2017, it had been found in 43 U.S. states and four Canadian states [23]. *Halyomorpha halys* causes significant economic losses on tree fruits, ornamentals, vegetables, and field crops and thus has arguably become the most important agricultural insect pest in North America [24]. Most of the economic damage has occurred in the mid-Atlantic region where severe losses to fruit (apples, peach) and vegetable (pepper, soybean, corn) crops were observed in 2010 [24]. The damage to apple plantations in this area was more than $37 million [25].

*Halyomorpha halys*’ rapid spread and adaptation in North America has been attributed to many factors such as the absence of natural enemies, large reproductive potential, cold tolerance, and increased survival due to climate change [25]. Its ability to feed on a large number and wide variety of plant hosts may have also been a contributing factor for establishment in diverse North American habitats. In native Asia, *H. halys* is known to feed on 106 plant species [25] whereas in North America, at least 169 host species are known including many fruits, vegetables, field crops, ornamentals [23].

The underlying genetic constraints or advantages of *H. halys*’ generalist herbivory which allow it to successfully adapt to a wide variety of hosts are not known. Although *H. halys* transcriptomes have been published earlier [26, 27], these studies focused on the generation of molecular resources or investigated life history traits (e.g. immunity, reproduction, development). Here, we generated a *H. halys* transcriptome to specifically investigate the diversity and characterization of P450 and cathepsin genes which, in turn, will provide useful insights into putative mechanisms of *H. halys*’ generalist herbivory. Enhancing further understanding into the generalist behaviour and various other aspects of basic biology in *H. halys* can provide additional knowledge for the success of invasive species and to develop sustainable pest management programs for agricultural crops.

## Methods

### Field collection and laboratory colony establishment

During summer 2012, *H. halys* adults were collected from a soybean farm at the Ohio Agricultural Research and Development Center (40^o^ 45′ 52″ N, 81^o^ 54′ 34″W, Wooster, OH, U.S.). A subset of *H. halys* adults was readily frozen for subsequent RNA extraction and cDNA library preparation (described below). To set up a laboratory colony, remaining *H. halys* adults were moved to inhabit the rearing cages (Catalog #1452, BioQuip Products, Rancho Dominguez, CA, U.S.). The rearing cages were maintained in a growth chamber at 28 ± 2 °C, 60–70% relative humidity, and 16:8 (light:dark) photoperiod. In the laboratory colony, the *H. halys* were fed with a mixed diet that included corn cobs, green beans, grapes, lettuce and carrots. Besides, standard rearing practices for *H. halys*, as suggested in [28], were adopted.

### RNA extraction and libraries preparation for RNA-Seq

Before processing for RNA extraction, both legs and wings from the frozen, field-collected *H. halys* adults were excised and discarded. Total RNA extraction was performed using PureLink® RNA Mini Kit (Life Technologies Corporation, Carlsbad, CA, US), as per the protocol provided by the manufacturer. In order to remove DNA contamination, RNA preparations were treated with PureLink® DNase (Life Technologies Corporation, Carlsbad, CA, U.S.). The Nanodrop 2000c (Thermo Scientific, Hudson, NH, U.S.) and an Agilent Bioanalyzer 2100 (Agilent Technologies, Palo Alto, CA, U.S.) were used to determine the RNA quality. High quality RNA preparations were processed to synthesize cDNA libraries using the TruSeq RNA Sample Preparation Kit (Illumina Inc., San Diego, CA, U.S.), as per the manufacturer’s protocol. Specific steps followed for library preparation are described in detail previously [29]. To facilitate subsequent demultiplexing of the read data, unique adapters were included for each cDNA library. To ensure high quality, the cDNA library samples were run onto a high sensitivity DNA chip using an Agilent Bioanalyzer 2100 (Agilent Technologies, Palo Alto, CA, U.S.). Pooled cDNA libraries were run on a HiSeq 2000 flow cell (Illumina Inc., San Diego, CA, U.S.) and were sequenced for single-end and paired-end reads. Sequencing was performed at the Core Facility, Ohio State University, Columbus, OH, U.S.

### Data processing, de novo assembly and functional annotation

In order to allocate RNA-Seq data to various samples, raw reads were demultiplexed using the respective index sequence. The raw data processing and de novo assembly construction were performed within the CLC Genomics Workbench version 6.5.1 (CLC Bio, Cambridge, MA, U.S.). Reads with either of the following characteristics were discarded: less than 40 bases of length; occurrence of one or more ambiguous/undetermined nucleotides; occurrence of fragments with a quality score below 0.01 (Phred score 20). For de novo assembly construction, word size of 24 and bubble size of 50 were selected. The assembly contigs with a length of less than 150 bases were discarded. To annotate the transcriptome, blastx program inbuilt within the Blast2GO platform was employed [30]. During the annotation, *H. halys* contigs were searched (*e* value <1.0E-3) against the NCBI Reference Sequence database (RefSeq protein). The blastx search was followed by mapping to gene ontology (GO) terms, and finally only the GO terms meeting the criteria of *e* value less than 1.0E-6, annotation score less than 55, and GO weight more than 5 were retained for annotation. The ‘Aqua’ method within ‘CateGOrizer’ tool was used to classify GO terms into different categories [31]. The GO categories obtained for *H. halys* transcriptome were compared to those from the *A. pisum*, available at [32]. The *H. halys* contigs lacking any significant hit to the RefSeq protein database were searched against non-redundant nucleotide (nt) database at NCBI using blastn (*e* value <1.0E-3) tool. To know the pathways in which putative proteins in *H. halys* are implicated, Kyoto Encyclopedia of Genes and Genomes (KEGG) database was searched against using Blast2GO [33]. The protein family (Pfam) domains in the transcripts were identified by searching against the Pfam database, integrated within the CLC genomics workbench (*E* value <1.0E-3) [34]. For comparative genomics, pairwise blastx searches (*E* value <1.0E-3) between *H. halys* transcripts and model insect genomes [*A. pisum*, *Bombyx mori* (silkworm), *Drosophila melanogaster* (fruit fly), *Nasonia vitripennis* (jewel wasp), *Rhodnius prolixus* (assassin bug), *Tribolium casteneum* (red flour beetle)] were performed. To calculate ortholog hit ratio (OHR), the number of non-gap characters in the query (*H. halys* transcript) were divided by the length of the subject (model insect ortholog) obtained during pairwise blastx search [35]. Assuming a conserved gene length between species, an OHR near zero suggests a poor assembly while value near one suggests a fully assembled transcriptome [35].

### Sequence analysis for P450 and cathepsin genes in *H. halys*

Classification and estimation for total gene counts for P450 and cathepsins were performed on the basis of their putative orthologs in *T. castaneum*. *Tribolium castaneum* was selected as a reference for sequence analysis because the majority of *H. halys* P450 and cathepsin transcripts had top hits to their counterparts in *T. casteneum*. Initially, nucleotide sequences for transcripts having top hit description ‘cytochrome P450’ and ‘cathepsin’ (or ‘cysteine peptidase’) (on blastx search to RefSeq database) were retrieved from the de novo assembly and were subjected to pairwise blastx search to *T. castaneum* proteins. If two or more *H. halys* transcripts had the same top hit in *T. castaneum*, their protein alignments were manually inspected. If either of the two *H. halys* transcripts had a complete open reading frame (ORF) or truncated ORF but with an overlap in their alignment to a *T. castaneum* protein, these were considered to have arisen from two different genes. Alternatively, transcripts with truncated ORFs that aligned to different regions of same *T. castaneum* protein without overlap were considered to be fragments transcribed from same gene, and were counted as only one in the final gene count estimates. Transcripts exhibiting ≥95% identity were considered isoforms arising from same gene. Subsequently, for the purpose of a total gene count of P450s and cathepsins, transcripts shorter than 250 bases were ignored to avoid overestimation. The preliminary draft of *H. halys* genome sequence has become recently available at GenBank (accession GCA_000696795.1) [36]. To obtain the CYP count, the *H. halys* genome database was searched with keyword ‘P450’. The search results were inspected manually to remove genes other than P450s; further any duplicates sequences were removed to avoid overestimation. The nomenclature for peptidases and their families was adopted as described in the MEROPS database [37].

### RT-PCR for P450 and cathepsin genes in *H. halys* tissues

Laboratory reared *H. halys* were used for tissue expression analysis of P450 and cathepsin genes. The *H. halys* female adults (5 day old) were dissected in phosphate-buffered saline (pH 8.0) under a dissecting microscope and the following tissues were obtained: salivary gland, gut, malpighian tubule, fat body, and ovary. Tissue samples were processed for RNA extraction and subsequent DNA-ase treatment as described in the previous section. The first strand cDNA was synthesized from DNA-free RNA samples using an iScript cDNA synthesis kit (Bio-Rad Laboratories, Hercules, CA, U.S.). The PrimerQuest design tool (Integrated DNA Technologies, Inc., Coralville, IA, U.S.) was used to design gene-specific primers (Additional file 1). Due to its stable expression, *EF1a* was used as internal control during RT-PCR [38]. Each RT-PCR reaction was performed 20 μl volume which contained 100 ng cDNA, 0.5 μM each of sense and antisense primers, and 10 *μ*l of PCR master mix (Promega Corporation, Madison, WI, U.S.). The cycling conditions for RT-PCR were as follows: one cycle of denaturation at 94 °C for 4 min, 33–40 cycles of denaturation at 95 °C for 30 s, annealing and extension at 55 °C for 30 s. The resultant PCR products were electrophoresed and visualized on a 2.0% agarose gel.

### Phylogenetic analysis

For P450s, a total of 112 amino acid sequences of CYP6 family in *H. halys*, *A. pisum*, *T. castaneum*, *R. prolixus*, and *D. melanogaster* were used. Only *H. halys* P450 transcripts encoding for a minimum of 150 amino acids in the ORF were included in this analysis. Further, *H. halys* P450 transcripts encoding for ORFs with non-overlapping N-terminal were not included in the analysis. Similarly, for phylogenetic analysis of cathepsins, a total of 108 (54 each for cathepsin-B and cathepsin-L) amino acid sequences from *H. halys*, *Riptortus pedestris* (bean bug), *A. pisum*, and *T. castaneum* were used. The transcript sequences of *H. halys* and GenBank accession numbers of protein sequences from other insects used in the phylogenetic analysis are given in Additional file 2. The phylogenetic analysis was performed in Mega7 software [39]. Before inferring the phylogeny, amino acid sequences were aligned through ClustalW (using default parameters) in Mega software. To infer the evolutionary history, the Maximum-Likelihood (ML) method (using all sites) was used. Models for ML analysis were selected with the analysis preference tool which suggested Le Gascuel (with frequency) [40] and Whelan and Goldman [41] as the best models for P450s and cathepsins analysis, respectively. To calculate the percentages of replicate trees in which sequences clustered together, a bootstrap test with 500 replicates was performed. To obtain the initial trees for the heuristic search, Neighbor-Join and BioNJ algorithms were applied to a matrix of pairwise distances calculated through a JTT model, and then a topology with superior log likelihood value (for cathepsins = −45,139.9392; for P450s = −29,087.84) was selected. To model the evolutionary rate differences among sites [5 categories; +G, parameter = 3.4001 (for P450s); 2.4928 (for cathepsins)], a discrete gamma distribution was used.

## Results and discussion

### De novo assembly

**Fig. 1:**
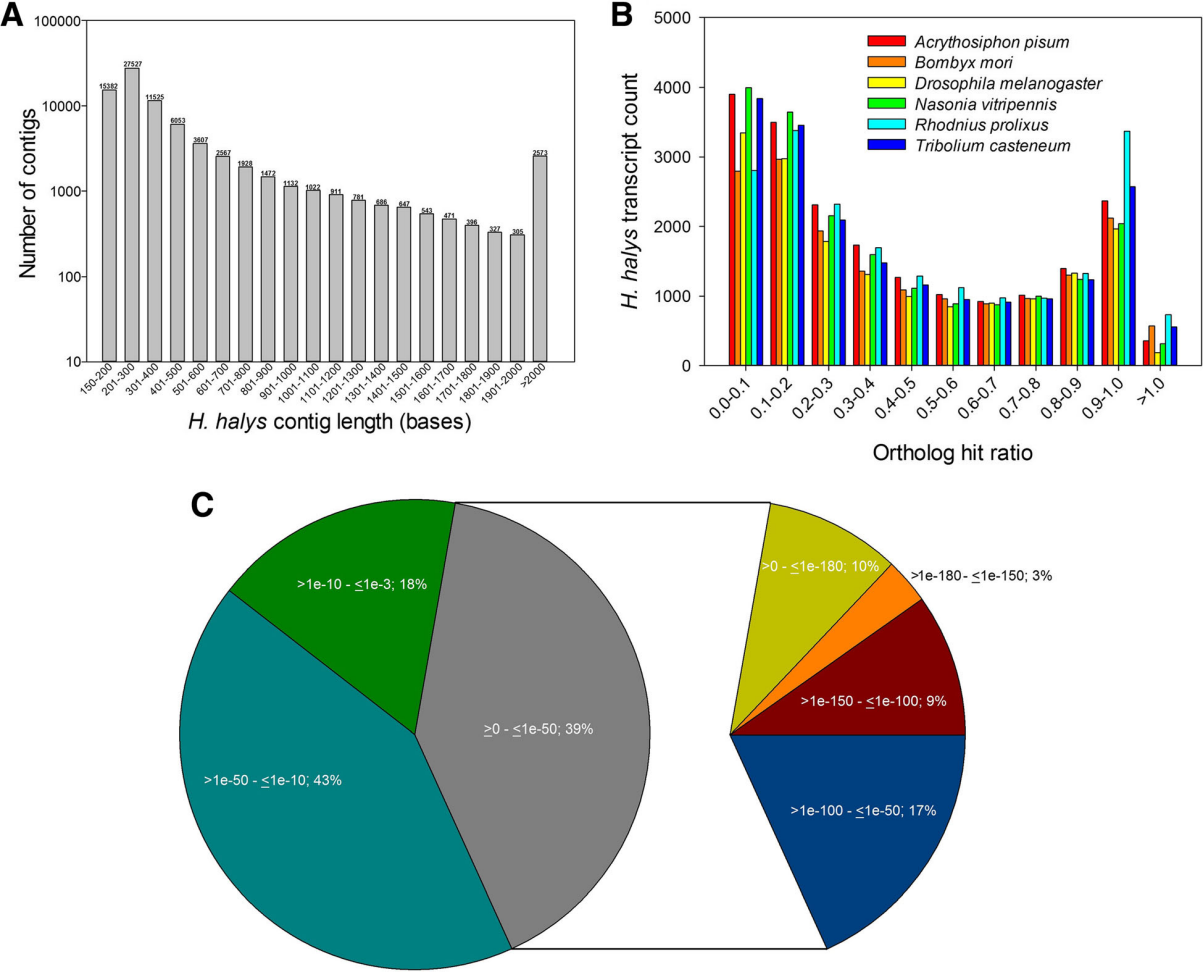
*Halyomorpha halys* de novo assembly. **a** Length distribution of 79,855 transcript contigs in de novo assembly (**b**) Ortholog hit ratio for transcripts calculated after blastx searches to genomes of *A. pisum*, *B. mori*, *D. melanogaster*, *N. vitripennis*, *R. prolixus*, and *T. castaneum* (**c**) Distribution of *E* values for top hits obtained during blastx search is shown

RNA-Seq for *H. halys* yielded a total of 142,856,464 high quality 50-bases single-end and 100-bases paired-end reads. The de novo assembly from *H. halys* RNA-seq data produced 79,855 high quality transcript contigs, totaling 39,600,178 bases. The length of transcripts in the assembly varied from 150 to 23,082 nucleotides with an average of 496 nucleotides. Nearly ~68% (54,434/79,855) of transcripts were less than 500 bases in length while a few (2573/79,855) exceeded 2 kb (Fig. 1a). The assembly’s N_50_ = 704 (the shortest sequence length at 50% of the transcriptome) was relatively high for a non-model organism. To estimate the completeness of transcriptome assembly, each *H. halys* transcript was compared to its putative ortholog in various model insects *A. pisum*, *B. mori, D. melanogaster, N. vitripennis, R. prolixus, and T. casteneum*. Overall, 26–32% of the transcripts (with matches) had an OHR > 0.7 and 36–43% had > 0.5 (Fig. 1b).

### Annotation and putative ortholog identification for *H. halys* transcripts

**Fig. 2:**
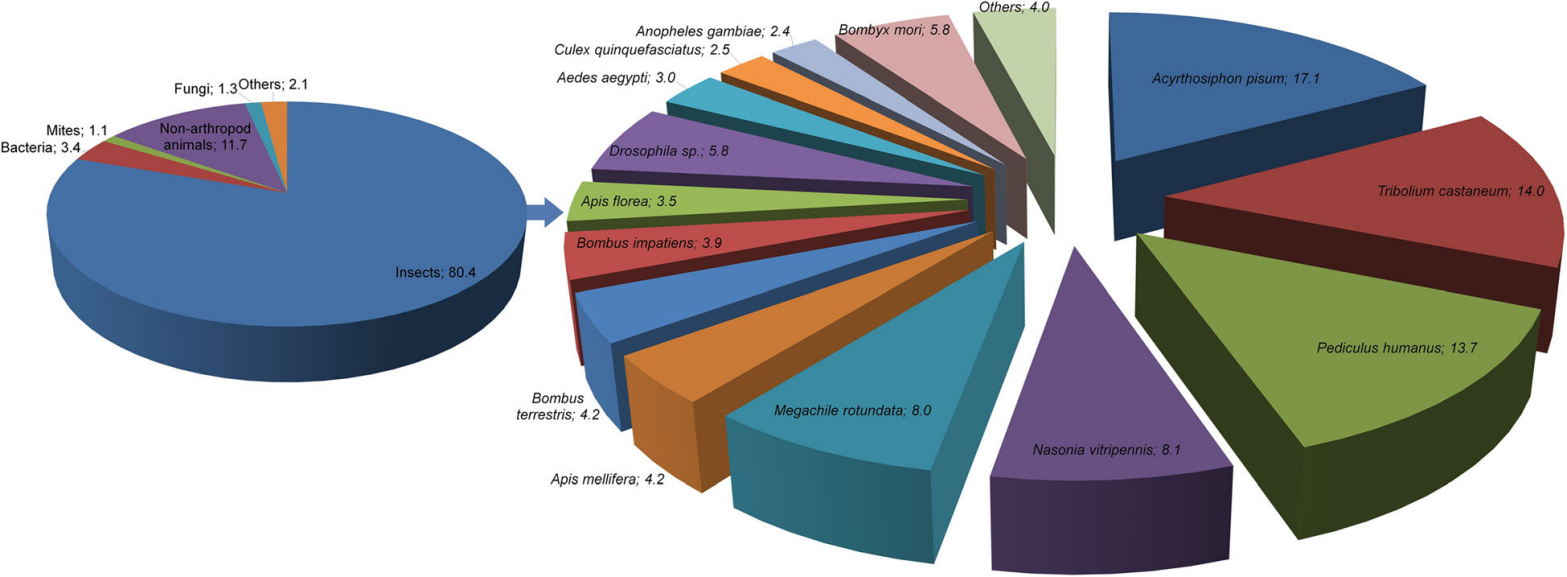
Summary of top hit organisms in blastx search for *Halyomorpha halys* transcripts. Overall distribution for the whole transcriptome is shown on the left whereas insect specific distributions are presented on right

The assembled transcripts were used as a query for a blastx search against the RefSeq protein database at GenBank. The e-value distribution for the best hits showed that 39% of transcripts have strong homology (e value ≤1e-50) (Fig. 1c). Furthermore, 10% of the transcripts had an extremely low e-value of less than 1e-180 which was rounded to zero. Overall, nearly 26% (20,772/79,855) of the *H. halys* transcripts had one or more hits (e value <1.0e-3) to protein sequences in the database (Additional file 3). A majority of top blast hits for *H. halys* transcripts were to insects (80.4%), whereas the rest were shared amongst non-arthropod animals (11.7%), bacteria (3.4%), mites (1.1%), and fungi (1.3%) (Fig. 2). A few groups such as protozoa, algae, archaea, and viruses constituted a minor fraction (1.2%) among top hits. As expected, the largest number of *H. halys* transcripts’ top hits was to *A. pisum* (17.1%), which was closely followed by *T. casteneum* (14.0%) and *Pediculus humanus* (13.7%) (Fig. 2).

A large proportion of *H. halys* transcripts (59,083/79,855) apparently had no significant match in RefSeq protein database, indicating many of them may be novel sequences or specific to *Pentatomidae* or *Halyomorpha* which are not well represented in the database. This was supported in the subsequent blastn search for the 59,083 unknown transcripts (found during blastx search) as hits for only 192 transcripts were recovered (Additional file 4). Interestingly, an InterProScan revealed hits to the protein signature domains for 15,811 out of 59,083 unknown transcripts (26.76%), suggesting that many have functional homologs (a Pfam search also revealed hits for unknown transcripts, see below) (Additional file 5). Nonetheless, the relatively high number of unknown transcripts found in current study was not surprising as similar results were obtained in earlier transcriptomic studies in *H. halys* and other non-model insects [26, 27, 29, 42, 43].

### Comparative genomics

**Fig. 3:**
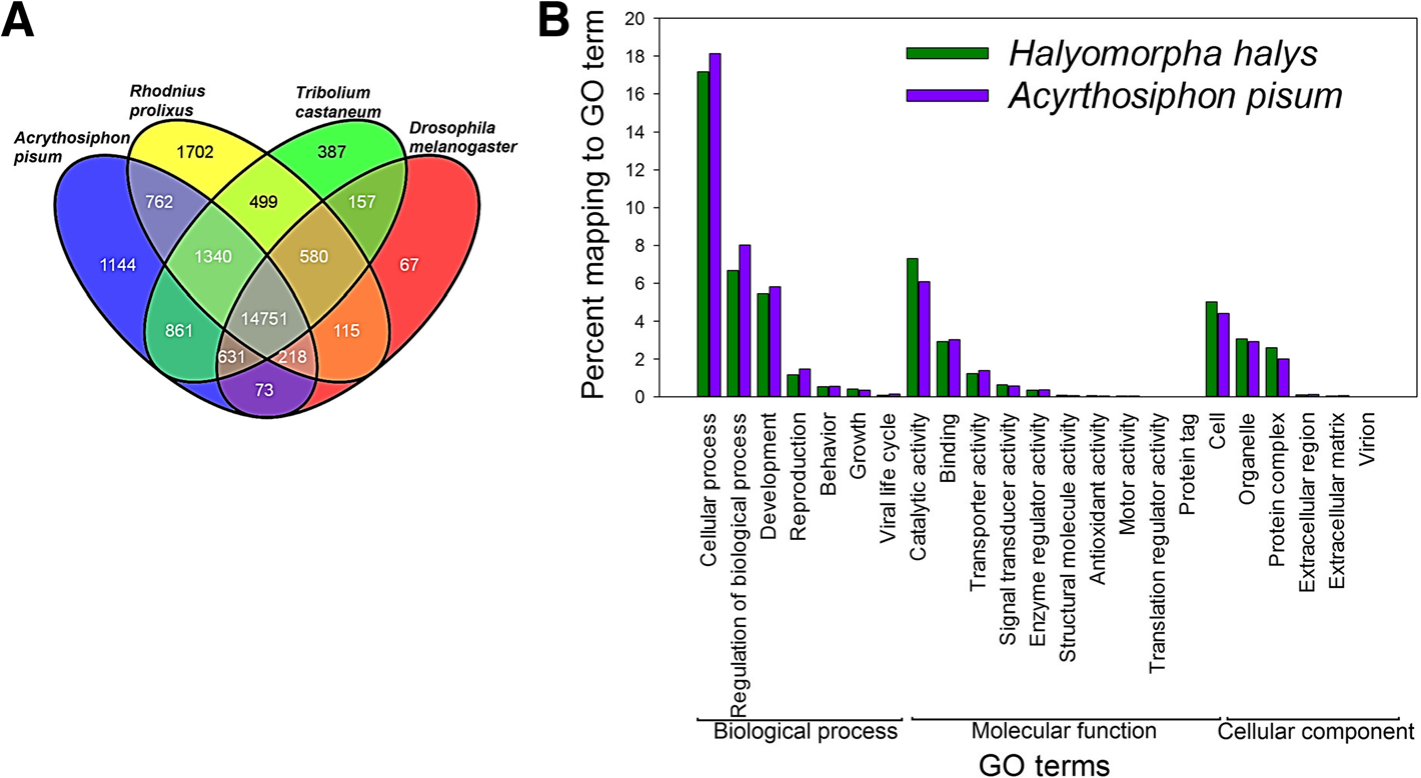
Comparative genomics for *Halyomorpha halys* transcriptome. **a** Venn diagram showing the number of transcript contigs with significant matches (unique and common) to genomes of *A. pisum*, *D. melanogaster*, *R. prolixus*, and *T. castaneum*. Significant matches (*e* value <1.0E-3) were calculated after pairwise comparisons (blastx) to each individual genome. **b** Comparison of GO term mappings distributions of *H. halys* and *A. pisum* that belong to each of the three top-level GO categories (i.e. biological process, molecular function, and cellular component)

We found significant hits for *H. halys* transcripts (combined = 23,287/ 79,855) upon pairwise blastx searches against protein databases of four model insects. A search into the *R. prolixus* database resulted in significant hits for highest number of *H. halys* transcripts (*n* = 19,967), which was closely followed by searches into *A. pisum* (n = 19,780) and *T. castaneum* (n = 19,206) databases. A large number of *H. halys* transcripts (*n* = 14,751) had hits to all the searched databases (Fig. 3a). Interestingly, the number of *H. halys* transcripts uniquely matching to hemipterans (i.e. *R. prolixus* (*n* = 1702) and *A. pisum* (*n* = 1144)) were not substantially high.

### Functional annotation: GO annotation, KEGG pathways, and Pfam domains

#### GO annotation

GO terms were assigned to 8951 *H. halys* transcripts (Additional file 6). The GO assignment resulted in 3160 biological process terms being assigned 21,071 times to 6330 *H. halys* transcripts, 671 cellular component terms being assigned 7788 times to 4463 transcripts, and 1466 molecular function terms being assigned 13,756 times to 7594 transcripts. A wide variety of GO terms from each of the three domains (biological process, molecular function and cellular component) was found to be assigned to *H. halys* transcripts (Additional file 6). ‘Regulation of transcription, DNA-dependent’ (424), ‘ATP binding’ (697), and ‘integral to membrane’ (722) were the most dominant biological process, molecular function, and cellular component terms, respectively. A comparison of percent mappings to GO categories revealed that both *H. halys* and *A. pisum* have similar distribution of mapped GO terms, which was not surprising as both are hemipteran species (Fig. 3b). However, the percentage of GO terms belonging to the ‘biological process’ domain encompassing cellular, regulatory, developmental, and reproductive activities was relatively lower in *H. halys* compared to *A. pisum*. Further, transcripts assigned to the ‘molecular function’ domain were predicted to encode for polypeptides with catalytic, binding, transporter, and signal transduction functions, with *H. halys* transcripts showing a slightly higher percentage (7.3%) of catalytic activity (GO:0003824) annotation compared to those of *A. pisum* (6.1%).

#### KEGG pathways

Using the KEGG-based pathway analysis, *H. halys* transcripts were predicted to be involved in one or more of 123 total pathways (Additional file 7). The putative proteins encoded from a large number of these transcripts were assigned to vital processes for nucleotide biosynthesis and metabolism such as purine (332 transcripts) or pyrimidine biosynthesis (89 transcripts). Interestingly, a total of 115 transcripts were linked to three related pathways: drug metabolism -other enzymes; drug metabolism - cytochrome P450; and metabolism of xenobiotics by cytochrome P450.

#### Pfam domains

A Pfam domain search yielded 44,637 domains in 11,354 *H. halys* transcripts (Additional file 8). Among the identified Pfam domains, ankyrin repeat (ANK-) domain (PF00023) was found to be the highest in occurrence with 5258 domains distributed within 634 transcripts. The recovery of such a large number of ANK-domains was not surprising as these are the most common structural motifs found in eukaryotic proteins [44]. ANK-domains consist of a tandem motif of ~33 amino acids with two alpha helices separated by loops and are known to mediate protein-protein interactions [44]. The ANK-domains are discussed more in section below.

The zinc finger (C_2_H_2_ type; PF00096) was the second most abundant Pfam domain found with 3613 domains distributed within 395 transcripts. In eukaryotes, zinc-finger proteins comprise the largest family of DNA-binding transcription factors which regulate gene expression during environmental stresses as well as other biological processes [45]. The WD domain, G-beta repeat (WD40; PF00400) and leucine rich repeat (LRR; PF00560) were among the other top Pfam domains identified in *H. halys* transcriptome. WD40 proteins are known to play a vital role in RNA processing, signal transduction, cytoskeleton assembly, cell division and protein- protein interactions [46] whereas LRR-proteins are involved in insect defence against biotic stresses [47].

Several domains linked to detoxification enzymes such as cytochrome P450 (PF00067; *n* = 256) and glutathione S-transferase (GST; PF02798; *n* = 54) were identified in *H. halys* transcriptome. In addition to P450s, GSTs are known to detoxify a wide-range of xenobiotics including PSMs and synthetic chemicals in several insect species [10]. Similarly, the carboxylesterase (CE; PF00135; *n* = 152) domain was found in a large number of transcripts. The CEs are general detoxification enzymes implicated in pest resistance to synthetic insecticides such as carbamates, pyrethroids and organophosphates [10]. However, the role of CEs in providing resistance to PSMs is not clear. Other highly abundant and common domains are listed in Additional file 8.

### Insights into *H. halys*’ generalist herbivory adaptation

One of the major factors in invasive *H. halys*’ successful adaptation in North America seems to be its generalist-feeding ability. Due to its interaction with multiple host plants, *H. halys* likely encounters several diverse challenges. In addition to facing a diverse diet, each containing a distinct nutrient composition, plant defenses such as directly toxic metabolites (e.g. PSMs), plant proteases and digestive inhibitors impact insect gut structures and enzymes. We focused our characterization on two gene families known to be involved in insect adaptation on host plants: cytochrome P450s and cathepsins.

### Expansion and tissue specific expression analysis for detoxifying P450s

**Fig. 4:**
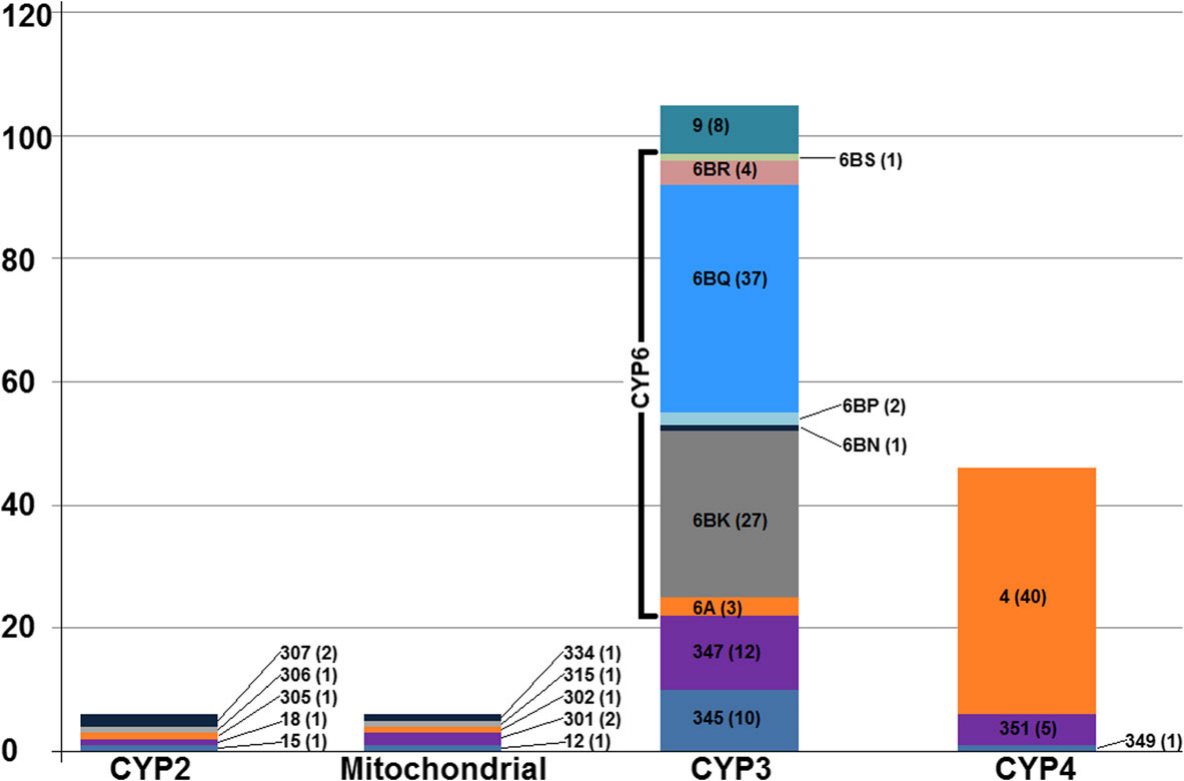
Classification of cytochrome P450 genes in *Halyomorpha halys.* Clan and family distribution of P450 genes in *H. halys* is shown. The number shown along each column represents the P450 family and the number in parenthesis is the number of individual genes predicted in the corresponding family

A blastx search using the RefSeq protein database revealed 223 transcripts in the *H. halys* transcriptome having top hits to insect P450s. Further analysis of these P450 transcripts based on *T. castaneum* P450s and subsequent size selection (>250 nucleotides, see Methods) indicated that these transcripts arise from a minimum of 163 CYP genes. An analysis into recently available genome sequence showed 161 uniquely encoded P450s so far (data not shown), thus our estimated CYP gene count based on the transcriptome is nearly identical to the one revealed in *H. halys* genome. A comparison of CYPome sizes for various arthropods showed that *H. halys* possesses one of the highest number of CYP genes (Table 1). These genes fell into all four CYP clans. Within the CYP clans, *H. halys* P450 genes were classified into a total of 17 families (Fig. 4). Among arthropods, genes belonging to CYP3 and CYP4 clans are usually in the highest proportion, and transcripts in these two clans seemed to be overrepresented in *H. halys.* Our data suggested 4 families, 10 subfamilies, 105 individual genes for CYP3 and 3 families, 7 subfamilies, 46 individual genes for CYP4 clan. The gene proliferation was notable especially in families 6 (75 individual genes) and 4 (40 individual genes) of CYP3 and CYP4 clans, respectively.

**Table 1:** P450 gene counts and their clan-wise distribution in various arthropods

Arthropod	Total^a^	Clan			
		CYP2	Mitochondrial	CYP3	CYP4
*Pediculus humanus*	36	8	8	11	9
*Apis mellifera*	46	8	6	28	4
*Acyrthosiphon pisum*	64	10	8	23	23
*Daphnia pulex*	75	20	6	12	37
*Drosophila melanogaster*	84	4	9	39	32
*Bombyx mori*	85	7	12	30	36
*Tetranychus urticae*	86	48	5	10	23
*Rhodnius prolixus*	88	5	6	50	27
*Nasonia vitripennis*	92	7	7	48	30
*Anopheles gambiae*	105	10	9	40	46
*Tribolium castaneum*	143	8	9	79	47
*Aedes aegypti*	160	12	9	82	57
Halyomorpha halys	≥163	6	6	105	46
*Culex quinquefasciatus*	204	16	12	89	82

^a^The ‘≥’ symbol indicates greater than or equal to

**Fig. 5:**
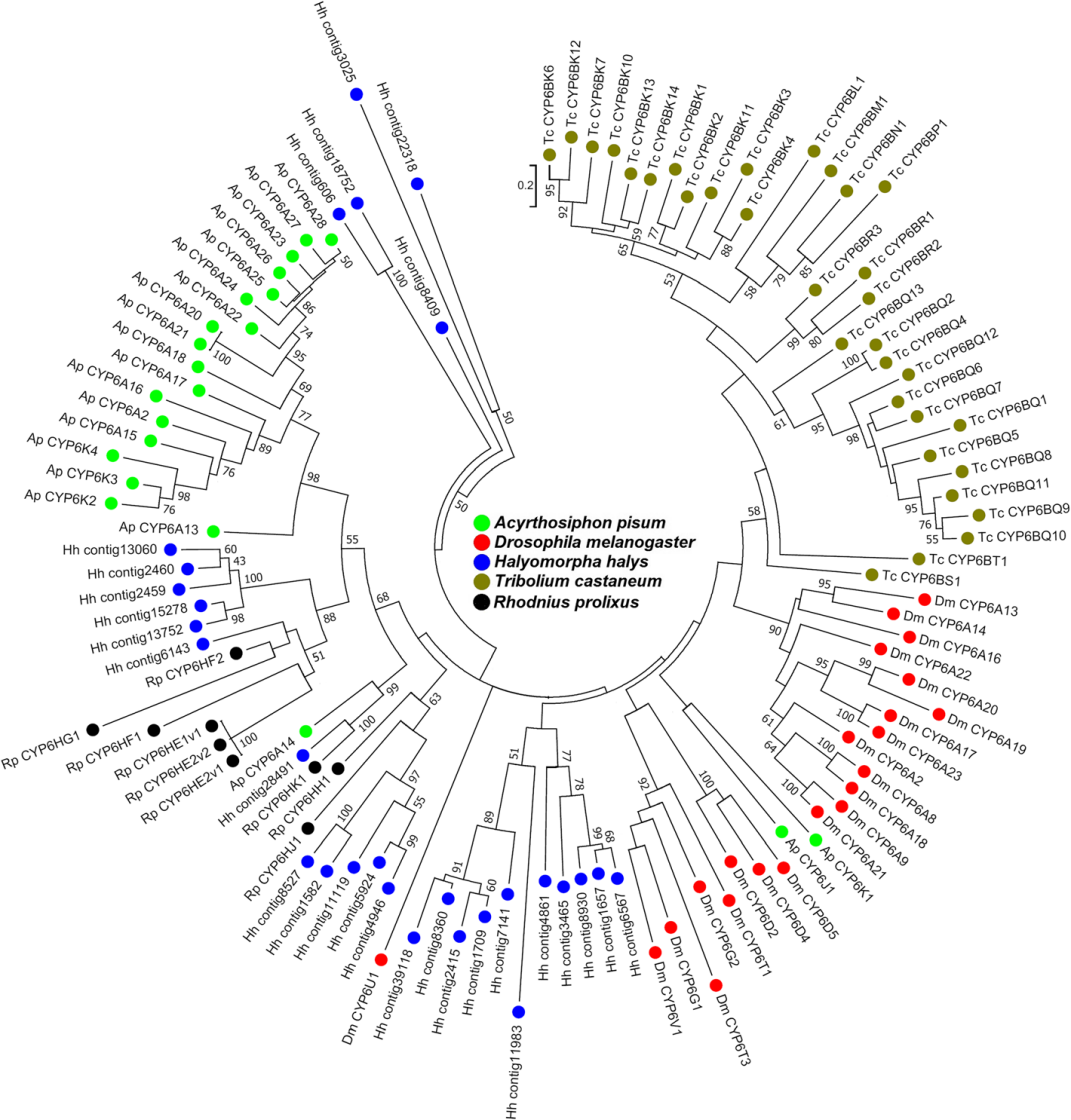
Phylogenetic analysis of P450s in *Halyomorpha halys.* A phylogenetic tree generated using P450s of CYP6 family in *H. halys* and other insects is shown. The evolutionary history was inferred by using the Maximum Likelihood method based on the Le Gascuel model. All nodes have significant bootstrap support based on 500 replicates. The bootstrap values only above 50% are shown next to branches. GenBank accession numbers for various protein sequences used in the phylogenetic analysis are provided in Additional file 2

CYP6’s are well known for their role in mediating insect-plant interactions [10] and constructing the evolutionary relationships of CYP6 family genes among insect CYPomes can provide evolutionary insight into their genetic divergence and function. We constructed a phylogenetic tree of *H. halys* CYP6s with corresponding genes identified from genomes of *T. castaneum*, *D. melanogaster*, *R. prolixus* and *A. pisum* (Fig. 5). Overall, the phylogenetic analysis showed a clustering of CYP6 genes respective of species, with the only exception of a putatively orthologous pairing seen between ApCYP6A14, RpCYP6HK1 and Hhcontig28491. Notably, the phylogenetic tree suggested a *H. halys*-specific expansion and sequence diversification of CYP6 genes. Although species-specific expansion involving gene duplication followed by divergence in CYP6 family genes is a characteristic feature in insect CYPomes [14], and has been documented in several insects [48–55], the extent of CYP6 gene expansion observed in *H. halys* appears to be substantially higher (Table 1 and Fig. 5).

The CYP6 family members, in general, are best known for their role in governing the ecological adaptation of insects by mediating metabolic resistance to a broader set of chemical challenges such as PSMs. In the *H. halys* transcriptome, the bulk of CYP6 transcripts (72/75) were CYP6B type as CYP6BQ (37 individual genes) and CYP6BK genes (27 individual genes) were the most abundant. The occurrence of large, diverse and amplified clusters of CYP6BQ and CYP6BK genes in the generalist *H. halys* is significant as several of these genes are implicated specifically in host plant adaptation. For example, in the generalists *Papilio glaucus* and *P. multicaudatus*, CYP6B enzymes mediate adaptation on various plants belonging to diverse families by detoxifying several types of linear and angular furanocoumarins encountered in their hosts [56–58]. Similarly, *Helicoverpa zea* feeds on hundreds of host plant species and different CYP6B enzymes mediate adaptation by detoxifying a wide range of PSMs including xanthotoxin, flavone, α-naphthoflavone, chlorogenic acid, indole-3-carbinol, quercetin, and rutin [59, 60].

**Fig. 6:**
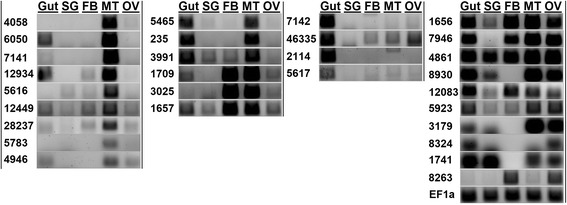
Tissue expression analysis for P450 genes in *Halyomorpha halys.* Results of semi quantitative PCR for expression analysis of CYP6BQ genes in *H. halys* gut, salivary gland (SG), fat body (FB), malpighian tubule (MT), and ovary (OV) tissues are shown (gel panels A-D; from left). Numbers on left for each gel section indicate P450 contig ID in the transcriptome assembly. *HhEF1a* was used as internal control. Primers and contig sequences are provided in Additional file 1

Not only does *H. halys* contain an expanded set of CYP6 genes, their expression is quite diverse among insect tissues based on semi-quantitative RT-PCR in different adult tissues (Fig. 6). Of the 30 *H. halys* CYP6BQ genes assayed, 9 showed expression predominantly in the malpighian tubules with minimal to no expression in other tissues (panel A, from left). An additional 6 transcripts showed high expression in the malpighian tubule but were also highly expressed in either gut or fat body (panel B). Overall, 20 out of 30 CYP6BQ gene assayed showed considerably high to peak levels of expression in malpighian tubules, which are traditionally regarded as the principal organs for excretion and osmoregulation in insects. However, it has recently been demonstrated that the malpighian tubules are also sites for the metabolic detoxification of PSMs and insecticides [61, 62].

Besides malpighian tubules, CYP6BQ genes were primarily expressed in the gut and fat body which is consistent with a presumed role of detoxification in these tissues. Four transcripts showed peak expression in the gut with minimal to no expression in other tissues (panel C) and the remaining transcripts were expressed in at least 3 or more tissues (except Hhcontig8263 which was expressed only in fat body and ovaries) (panel D). These data indicate that *H. halys* exhibits a diverse and varied expression pattern of its suite of CYP6 P450s, and is consistent with evolutionary analysis suggesting that CYP6B genes governing host plant adaptation and differential tissue level expressions are sub-functionalized (after the gene duplication) [63]. However, further functional studies such as protein expression/induction and substrate activity are needed to provide definitive evidence on the evolution of CYP6B genes in *H. halys*.

### Expansion and gut specificity of cysteine peptidase L-like (cathepsin-L) genes

**Fig. 7:**
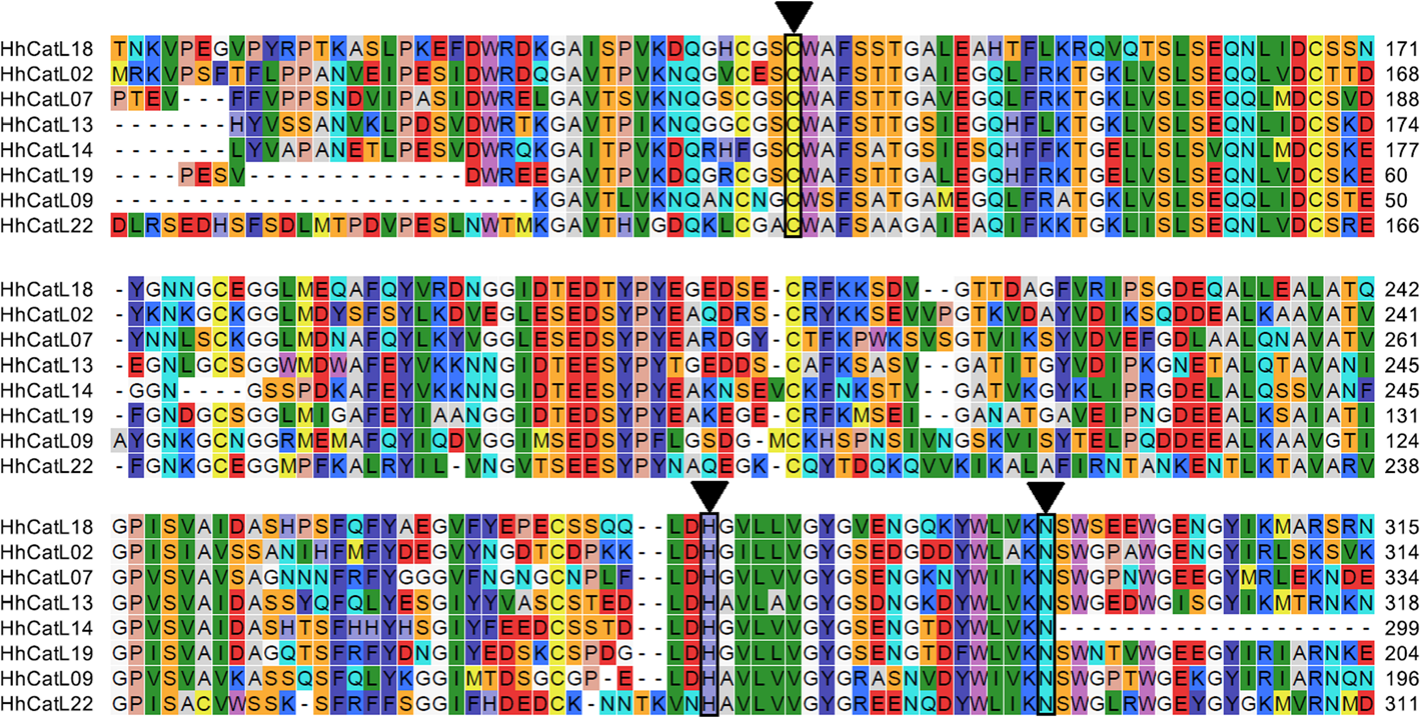
Amino acid alignment of cathepsins in *Halyomorpha halys.* Amino acid alignment of a few selected cathepsin-L proteases is shown. Functionally important residues C, H, and N (active sites) are boxed (indicated by arrows)

Due to acidic nature of the gut (pH ~5.5), heteropterans rely upon cysteine peptidase-like cathepsins to carry out their gut proteolytic activities, as opposed to serine peptidases-like cathepsins [which are active in the neutral/alkaline gut of other insects] [64]. Therefore, to gain insights into mechanisms underlying generalist herbivory, we focused on *H. halys*’ cysteine peptidase-like cathepsin genes. In the *H. halys* transcriptome, we identified a total of 124 transcripts encoding for cysteine peptidases which were classified to belong to four different families: C1 (papains), C2 (calpains), C13 (legumains), and C14 (caspases). Within the C1 family, we found 20 transcripts for cathepsin-B and 67 transcripts for cathepsin-L which, in turn, were predicted to be transcribed from at least 10 and 33 genes, respectively (Table 2). A comparison of cysteine peptidase family genes among different insects suggested that the cathepsin-L gene count observed in *H. halys* was the highest known so far (Table 2). Multiple sequence alignment for putative cathepsins revealed the conservation of three key amino acid residues in cysteine proteases which are cysteine (C), histidine (H), and asparagine (N) at the active site (Fig. 7).

**Table 2:** Number of genes for different cysteine peptidase families found in insects

Cysteine peptidases^a^	*Drosophila melanogaster*	*Tribolium castaneum*	*Apis mellifera*	*Bombyx mori*	*Acyrthosiphon pisum*	*Halyomorpha halys* ^bcd^
C1A (Papain): Cathepsin-B	1	11	1	1	34	≥10 (20)
C1A (Papain): Cathepsin-L	9	13	1	2	2	≥33 (67)
C2 (Calpain)	4	7	4	5	8	≥11 (18)
C13 (Legumain)	0	0	0	0	5	7 (7)
C14 (Caspase)	7	8	5	4	6	≥9 (10)

^a^Cysteine peptidase counts for each insect are based on latest gene annotation from Genbank, as assessed on 07/02/2017

^b^The ‘≥’ symbol indicates greater than or equal to

^c^Figures in parentheses indicate total number of transcripts for a given gene family identified in *H. halys* transcriptome

^d^There was single transcript each for Cathepsin-J and Cathepsin-O identified in *H. halys* transcriptome, which are not indicated here

**Fig. 8:**
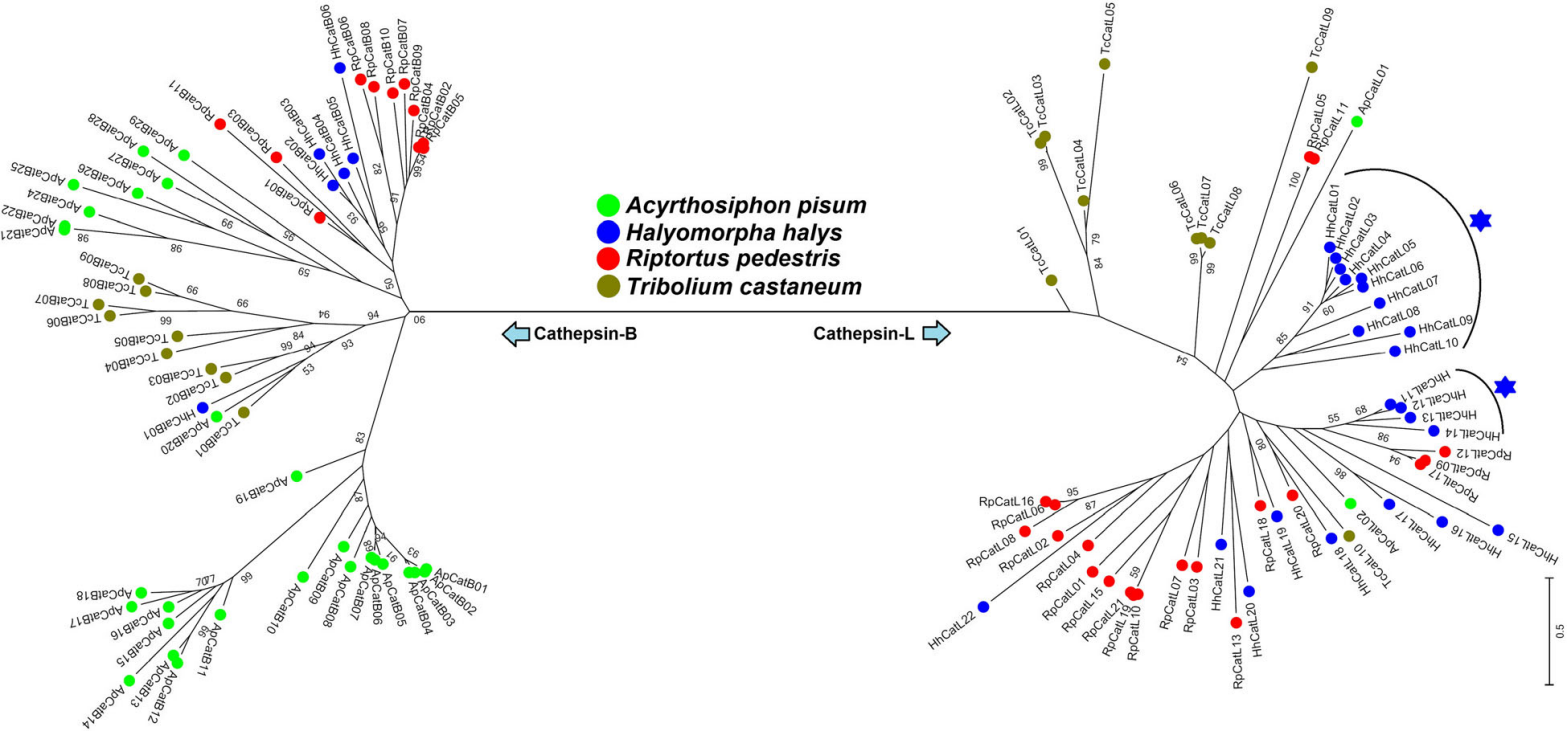
Phylogenetic analysis of cathepsins in *Halyomorpha halys****.*** A phylogenetic tree generated using cathepsins (Cathepsin-B and Cathepsin-L) in *H. halys* along with corresponding cathepsins in other insects is shown. The evolutionary history was inferred by using the Maximum Likelihood method based on the Whelan and Goldman model. All nodes have significant bootstrap support based on 1000 replicates. The bootstrap values only above 50% are shown next to branches. The asterisks indicate the locations of *H. halys* cathepsin-L gene expansion. GenBank accession numbers for various protein sequences used in the phylogenetic analysis are provided in Additional file 2

Phylogenetic analysis of *H. halys* cathepsins combined with other insects separated the cathepsin-B and cathepsin-L sequences into two distinct clusters (Fig. 8). A strong *H. halys*-specific expansion and sequence diversification was observed, especially in HhCatL01–10 and HhCatL11–14 cathepsin-L genes. We found very few examples of recognizable orthology of cathepsins among all four species in our comparison. Gene duplication and expansion of gene families appears to be a feature of hemipteran insects [65]. The cathepsin genes in particular have shown massive amplification: 13 cathepsin-B and 21 cathepsin-L transcripts have been found in the *R. pedestris* transcriptome [66], and similarly, 34 cathepsin-B and 2 cathepsin-L are found in *A. pisum* [67]. Our phylogenetic analysis supports the assertion that cathepsin genes have expanded in the stinkbug lineage and in the aphid lineage independently (Fig. 8) [66]. Further, both *H. halys* (Heteroptera: Pentatomidae) and *R. pedestris* (Heteroptera: Alydidae) belong to the infraorder Pentatomomorpha within Hemiptera. Although there were rare instances of orthology for cathepsin-L genes between *H. halys* and *R. pedestris*, overall the cathepsin-B and -L genes of *H. halys* formed clusters distinct from those of *R. pedestris* (Fig. 8). Thus, the expansion of cathepsin genes could be a genus or species-specific event in Pentatomomorpha lineages. Additional molecular data for other stink bug species, including predatory species such as *Podisus maculaventris*, would help understand cathepsin evolution in the Pentatomidae.

**Fig. 9:**
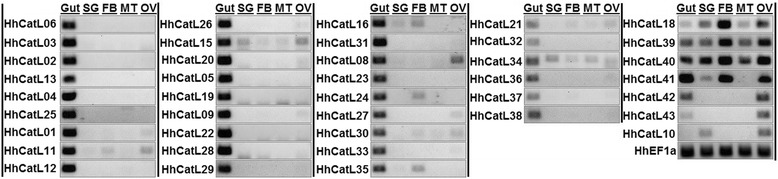
Tissue expression analysis for cathepsin-L genes in *Halyomorpha halys.* Results of semi quantitative PCR for expression analysis of cathepsin-L genes in *H. halys* gut, salivary gland (SG), fat body (FB), malpighian tubule (MT), and ovary (OV) tissues are shown (gel panels A-E; from left). *HhEF1a* was used as internal control. Primers sequences are provided in Additional file 1

Based on their role in a particular insect body compartment, cathepsins are known to exhibit a tissue-specific or distinct expression patterns. Thus, to identify candidate genes involved in *H. halys*’ interaction with and adaptation on host plants, we assessed the relative expression levels of cathepsin-L genes in different adult tissues including the gut which is at the direct molecular interface of plant-insect interactions (Fig. 9). Similar to P450’s we observed diverse cathepsin-L expression patterns. Out of 40 transcripts analysed, 33 have almost exclusive gut-specific overexpression compared to other tissues (Fig. 9 gel panels A-D, from left). Amongst these, 27 transcripts showed peak levels in gut (gel panels A-C) whereas 6 were expressed in relatively lower levels (gel panel D). In several stink bug and hemipteran species, cathepsin-L genes have showed gut expression not only at RNA level but also at protein level by exhibiting activities in functional enzymatic assays [20, 64, 66, 68]. Therefore, the gut specific expression for majority of cathepsin-L genes strongly supports their principal roles of defense against plant proteases/ protease inhibitors and the proteolytic digestion in *H. halys*. Further, the gut specific expression of a large number of cathepsin genes in *H. halys* is significant as insects are known to adapt to host plants by regulating expression of these genes in various ways [17]. Although our data suggested that such regulation is occurring, additional experiments are needed to test their expression on different diets, life stages, and in various environments.

## Conclusions

Our investigation into *H. halys*’ P450s and cathepsin-L peptidases, the principal components for host plant adaptations suggested considerable expansions in their gene repertoire. Expression data among different tissues for these genes were highly consistent with their purported functions of detoxification and digestion within the *H. halys* body. Expansions in P450 and cathepsin genes most likely reflected the generalist herbivory adaptations which enabled *H. halys* to overcome challenges such as toxic PSMs, digestive inhibitors, and the diverse and chemically rich diet encountered in its numerous host plants.

The extreme generalist behavior of *H. halys* likely aided in its successful establishment in North America. While data suggest that *H. halys* suffered a genetic bottleneck during invasion [22], the expanded and diverse set of P450’s and cathepsin discovered here may have helped overcome the lack of genetic diversity and the invasion paradox. If so, we might expect different and divergent expression patterns among native and invasive *H. halys* populations—a hypothesis that would add to our knowledge of adaptation during invasion induced stress. There is an additional possibility that *H. halys* employs transcriptional plasticity in regulating the expanded detoxification and digestive pathways to overcome diverse host-plant challenges [69]. However, these experiments include logistical and regulatory challenges, requiring the collection and transport of live insects from different countries. Nonetheless, future research elucidating the transcriptional behavior for detoxification and digestion during differential host feeding in *H. halys* will not only help understand the evolution of host breadth, but can also provide useful information for pest management.

## Additional files

Additional file 1: Primer sequences for RT-PCR of cathepsin-L and P450 genes in *Halyomorpha halys (XLSX 14 kb)*

Additional file 2: The GenBank accession numbers or transcripts of various protein sequences used in the phylogenetic analysis of cathepsins and P450s. (XLSX 38 kb)

Additional file 3: Putative ortholog and annotation details for *Halyomorpha halys* transcriptome assembly (XLSX 5983 kb)

Additional file 4: Results of blastn search performed on *Halyomorpha halys* transcripts having no match during blastx search to RefSeq database (XLSX 1989 kb)

Additional file 5: Results of InterProScan search performed on *Halyomorpha halys* transcripts having no match during blastx search to RefSeq database (XLSX 2047 kb)

Additional file 6: Gene ontology assignments for *Halyomorpha halys* transcriptome assembly. This file contains four spreadsheets. The “Gene Ontology” spreadsheet lists all three domains combined for each transcript whereas remaining three lists each domain (i.e. biological process, molecular function, and cellular component) separately. (XLSX 2529 kb)

Additional file 7: Putative KEGG pathways assignments for *Halyomorpha halys* transcripts (XLSX 61 kb)

Additional file 8: Predicted Pfam domains in *Halyomorpha halys* transcripts. This file contains two spreadsheets. The “Detailed” spreadsheet contains putative Pfam assignments to individual *H. halys* transcripts, while the “Summary” spreadsheet lists a summary of the counts of all Pfams identified. (XLSX 3871 kb)

## Abbreviations

CE: Carboxylesterase
GO: Gene ontology
GST: Glutathione S-transferase
KEGG: Kyoto Encyclopedia of Genes and Genomes
LRR: Leucine rich repeat
OHR: Ortholog hit ratio
ORF: Open reading frame
Pfam: Protein family
PSM: Plant secondary metabolite
